# **ERP Signals During Speech Articulation: Does Auditory Feedback Mask Other Ongoing Cognitive-motor Processes?**

**DOI:** 10.1007/s10548-025-01131-0

**Published:** 2025-08-14

**Authors:** Michael De Pretto, Ina Kodrasi, Marina Laganaro

**Affiliations:** 1 Faculty of Psychology and Educational Sciences, University of Geneva, Geneva, Switzerland; 2 Signal Processing for Communication Group, Idiap Research Institute, Martigny, Switzerland

**Keywords:** Speech production, Overlapping signals, ERP subtraction, Multi-channel wiener filter

## Abstract

The study of speech planning/programming may require analysing Event-Related Potentials (ERPs) during articulation. However, ERPs identified during speech production also contain brain signals associated with auditory feedback. Because these processes are both time-locked to the vocal onset, existing algorithms for signal separation have difficulties distinguishing one from the other. Here, we investigated the use of the multi-channel Wiener filter (MWF) to remove the ERP signal related to auditory processing from the ERP signal related to speech production. In a first step, participants were asked to overtly produce mono- or disyllabic pseudowords. In a second step, they had to listen to the recording of their own productions. We used the ERP signal associated with hearing one’s own production (referred to as listening ERPs) to estimate and filter out the auditory part of the production ERP. We investigated three versions of the ERPs during speech articulation: (1) the original production ERPs, (2) the ERPs filtered through the MWF, and (3) subtraction ERPs obtained by subtracting the listening ERPs from the original ERPs. Firstly, we conducted a microstate analysis comparing all three versions of the ERPs. Secondly, we conducted separate microstate analyses comparing mono- versus disyllabic conditions on each of the three versions. The results indicate that the subtraction method alters topography consistency and may remove relevant signal from the ERPs. The filtered ERP produced similar results to the original production ERP and thus, it may be unnecessary to remove auditory feedback or other overlapping signal if time-locked to the production onset.

**Supplementary Information:**

The online version contains supplementary material available at 10.1007/s10548-025-01131-0.

## Introduction

Producing speech requires encoding speech goals and motor programs, a cognitive-motor process that transforms a linguistic message into articulation (Guenther 2016). While it is generally accepted that motor speech encoding involves retrieving stored units larger than a phoneme (with syllabic units being a good candidate, e.g. Cholin et al. 2006; Laganaro and Alario 2006), which are then transformed into muscle-specific motor programs (Bohland and Guenther 2006; Parrell et al. 2019), it remains unclear whether planning/programming of multisyllabic utterances occur in full before production begins, or whether syllables are processed sequentially during articulation. Thus, in order to study the dynamics of speech planning/programming of multisyllabic utterances, it may be necessary to analyse Event-Related Potentials (ERPs) *during* articulation, as motor speech encoding may continue during the articulation of the first syllable.

Electrophysiological studies of speech production that analyse the signal during articulation are scarce, with most studies focusing on the pre-vocal onset signal. These studies have shown amplitude modulations depending on the complexity of the task over the speech Bereitschaftspotential component (Chandregowda et al. 2020; McArdle et al. 2009; Wohlert 1993). This component, observed at vertex sites, is considered to represent motor preparation and peaks around motor onset. The study by Bullock and collaborators (2025) suggest that at vocal onset (producing the word ‘scrambled’), the activation of the supplementary motor area, involved in motor planning, gives place to that of the premotor and primary motor cortices. It remains to be defined whether these areas or others are involved differently while producing mono- versus disyllabic utterances.

Speech planning and programming during articulation involve brain processes associated with producing speech overlapped with both motor artefacts and brain processes associated with auditory feedback, that is, the fact of hearing oneself while speaking (Parrell and Houde 2019). The aim of the current study was to test different options to remove the influence of auditory feedback brain signal from the speech production signal, and to assess whether the brain signal associated with auditory feedback may impact the analysis of speech production.

Surprisingly, while the impact of articulation on ERPs has been often considered and artefact cleaning procedures have been devised (e.g., Ouyang et al. 2016), the impact of auditory feedback in the study of the neural processing associated with speech planning and articulatory production has received, to our knowledge, little to no attention. Auditory feedback has been investigated as a phenomenon of interest (e.g., Li et al. 2024; Shiller et al. 2020), but not in terms of the impact of the associated cortical auditory evoked potentials (AEPs) on the signal of other cognitive processes running in parallel. For example, while uttering multisyllabic words, it may be that the AEP components associated with the production of the first syllable could mask processes associated with the planification of the second syllable.

EEG motor artefacts and other extra-cerebral artefacts are classically removed with appropriate filtering and averaging across trials, or ultimately by rejecting problematic epochs (Luck 2014). Additional advanced procedures can be applied for a more thorough cleaning of the signal (Delorme and Makeig 2004; Mullen et al. 2015). These procedures are especially useful when the number of artefact-free epochs is insufficient for removing the underlying noise by simple averaging. For example, independent component analysis (ICA) is one of the most popular EEG signal decomposition methods, typically used for cleaning artefacts associated with eye-blinks or muscle activity. Even though the efficacy of ICA for artefact removal has been demonstrated (Delorme et al. 2007; Jung et al. 2000a; Jung, Makeig, Westerfield, Jung et al. 2000a, b), it remains a debated procedure as it may remove more from the signal than the targeted artefacts (Pontifex et al. 2017). Principal component analysis (PCA) is another powerful tool for EEG artefact rejection as well as ERP analysis. However, PCA has showed limitations in the case of overlapping and/or highly correlated ERP components (Dien 1998; Scharf et al. 2022; Wood and McCarthy 1984). Most importantly, PCA cannot be used to target the removal of a specific signal because it is an unsupervised technique that relies solely on maximizing variance, without distinguishing between desired and undesired components.

Other algorithms may be used for cleaning artefacts (e.g., Chang et al. 2018; De Clercq et al. 2006; Ouyang et al. 2016; Vos et al. 2010). However, recent publications promote minimizing interventions on the signal (Delorme 2023). Notably, studies have shown that different artefact cleaning algorithms may lead to different results (Robbins et al. 2020). In other words, it is never entirely clear to what level these artefact cleaning algorithms may affect the signal of interest, and thus, that the choice of a cleaning algorithm may impact the derived conclusions. Consequently, artefact rejection and trial averaging may be the safest way of obtaining clean ERPs. Moreover, artefact rejection is robust to the method used for identifying bad epochs, whether manually or automatically (Malafeev et al. 2019; Shirk et al. 2017; Wu et al. 2018). It should be noted however, that averaging may blur the ERP components, especially components of later latencies. Typically, these are wider and have less identifiable peaks.

When it comes to distinguishing signals associated with different brain processes, the use of specific algorithms is almost inevitable. Indeed, when the processes to discriminate are not time-locked to each other, such as stimuli presentation and motor response (e.g., button press), the variability of the delay may not be large enough for the averaging to remove the overlapping signals that are not of interest. Algorithms developed for such cases have shown interesting results (e.g., Ehinger and Dimigen 2019; Ouyang et al. 2016). However, in the case of speech production and auditory feedback, the processes are both time-locked to the vocal onset, complexifying the separation of the signals.

In a similar context, Ross and collaborators (2022) tested a solution based on ICA for distinguishing AEPs induced by transcranial magnetic stimulation (TMS) from the signal of interest. By combining EEG recordings during TMS and sham TMS (i.e., when the noise of the TMS is produced without the magnetic stimulation), they could identify a component representing the AEP and subtract it form the signal. Compared to the subtraction of ERPs, the subtraction of an ICA components representing AEPs would theoretically take into account differences in AEP amplitudes across trials, and thus adjust to the inhibition of the auditory cortex while speaking. However, using ICA for distinguishing cognitive processes has been criticized for the same reason as for artefact rejection, and also due to the probable lack of independence of the brain sources (Michel et al. 2009; Ouyang et al. 2011). In the case of TMS, the sounds produced during stimulation and sham conditions are the same, and it may be argued that the brain sources of these AEPs are independent of the signal of interest.

Regarding speech production, the brain sources associated with motor speech encoding and with auditory feedback are unlikely to be independent. Auditory feedback plays a role in monitoring ones speech production and is thought to take place within the primary auditory cortex (Guenther and Hickok 2015). Hearing a sound is associated with strong AEP components around approximately 50 to 200 ms post-sound onset (Legatt 2015). These early cortical components are considered to represent mainly bottom-up sensory processing (Gommeren et al., 2021; Lunardelo et al., 2021; Paulraj et al., 2015). Thus, the presence of AEPs during speech production may mask ERP components related to the cognitive-motor planning/programming of upcoming speech. More specifically, it may mask differences associated with planning speech in different conditions, as for instance planning short versus longer speech sequences. In the current study, one of our aims was to assess the impact of AEP while producing mono- versus disyllabic pseudowords. More specifically, our objective was to investigate whether we could reveal masked differences between mono- or disyllabic word production by removing the auditory part of the production ERP within the time-window of the articulation of the first syllable.

**Possible Approaches To Remove AEPs during Articulation**: A simple solution to remove the AEP signal bound to be generated during speech articulation would consist of subtracting the AEP associated with auditory feedback from the speech production signal. This method is based on the assumption that the EEG scalp field represents the addition of the signal from various brain sources (Michel et al. 2009). For example, subtraction has been used to assess processing of body-related information when seeing images of bodies/actions, and by subtracting the signal from visual only trials (Galvez-Pol et al. 2020). A second assumption to use such a method is that the signal to subtract, recorded in isolation, is the same as its counterpart in the combined signal from which it will be subtracted (or is at least a very accurate estimate of it). This assumption seems reasonable for sensory ERPs, even though some top-down processes might influence these components (Mendel and Goldstein 1969).

In the case of auditory feedback during speech production, the recording of auditory feedback signal is impossible without speech production. As an alternative, the productions of the participants may be recorded and played back to the participants in a second step. However, hearing a recording of oneself is different from hearing oneself while speaking. Evidence suggest that the auditory cortex is partially inhibited during speech production, potentially reducing the amplitude of the AEPs (Guenther and Hickok 2015; Tremblay and Sato 2024). Moreover, speech auditory feedback is not only heard via air vibrations, but also via bone conductance (Maurer and Landis 1990), which is why people hardly recognise their own voice on recordings. For these reasons, subtraction of the AEP recorded as the participant listens to their own production is likely inappropriate for removing the AEP associated with auditory feedback during speech production.

To relax this restrictive assumption behind the subtraction method, that is, that the AEP recorded in isolation is the same as its counterpart during speech production, in this study we also considered a linear statistical filtering technique known as the multi-channel Wiener filter (MWF). The multi-channel Wiener filter has been widely used in the audio and speech domain to estimate a signal of interest in the presence of noise (Chen et al. 2006). The MWF has proven to be a highly adaptable and effective method for removing artifacts from EEG signals, demonstrating reliable performance across a wide range of applications and recording conditions. In their seminal work, Somers and collaborators (2018) introduced a versatile MWF-based algorithm that operates without requiring prior knowledge of the artifact sources. By utilizing statistical techniques to detect contaminated segments, this method applies adaptive spatial filtering to isolate and suppress artifacts while preserving the EEG signals of interest, showing notable effectiveness in removing diverse artifact types, including eye movements and muscle activity.

Expanding on this foundation, Asogbon and collaborators (2021) incorporated the MWF into a pipeline designed to sequentially eliminate multiple types of artifacts in motor imagery tasks. This integration significantly improved classification accuracy in motor imagery decoding. Rossi and collaborators (2024) adapted MWF to address the challenge of cochlear implant (CI) artifacts in paediatric EEGs. Their findings indicate that MWF effectively reduces CI-related noise without compromising EEG reliability. Finally, Bailey and collaborators (2023) further demonstrates the ability of the MWF to efficiently handle large datasets while preserving neural oscillations and removing a wide variety of artifacts.

Across these studies, a common assumption is that the artifacts present in EEG signals are spatially and temporally distinct from the neural activity of interest. We apply the same assumption to AEPs, where they are considered spatially and temporally distinct from speech production ERPs. In contrast to PCA for example, the MWF is a supervised method that explicitly models the covariance of both components, allowing it to selectively enhance the desired component while targeting and reducing specific undesired components based on their statistical properties. Thus, the MWF is deemed an appropriate tool for their suppression in such a scenario.

In summary, we compared two ways of removing the interference of auditory brain processing during speaking from the ERP signal related to speech planning and programming: (1) the linear filtering method; (2) the AEP subtraction method. We initially planned to also include the ICA method, but this method was inconclusive to identify AEP components during production. In a delayed production task, participants were asked to overtly produce mono- or disyllabic pseudowords. In a second part, they had to listen to the recordings of their own productions. Our main research question was whether the linear filtering method would reveal differences between conditions that would be absent from the comparison using the original production signals.

## Methods

### Participants

Thirty French native speakers participated in the study. All had normal hearing, normal or corrected to normal vision, and no history of language, speech, neurological or psychiatric disorders. They were all right handed following the Edinburgh Handedness Inventory (Oldfield, 1971). Before participating in the study, they provided written informed consent and were rewarded for their participation. The study was approved by the local ethics committee and followed the requirements of the Declaration of Helsinki. Two participants were excluded for not following the instructions (spoke between trials), and one was excluded due to a technical problem with the audio recording device. Furthermore, ten additional participants were discarded from the analyses, due to either poor EEG signal quality (see section *EEG acquisition and pre-processing*) or a high number of contaminated epochs in the ERP data. As a result, the study includes the remaining seventeen participants (12 females, mean age: 23.6 years old, range: 20–31 years old).

### Materials

Stimuli consisted of monosyllabic and disyllabic pseudowords. All of them were phonotactically legal sequences in French, and void of any meaning in order to remove any impact of lexical and semantic processes. Monosyllabic pseudowords consisted of seven CCV (consonant-consonant-vowel) syllables (/kRa/,/kRou/,/kla/,/pRa/,/pRu/,/plu/,/tRa/). Disyllabic pseudowords were created with a first CCV syllable that was the same as for monosyllabic stimuli to which a second CV syllable was added. Sixty disyllabic items were created (see Appendix A1). For each item, all consonants were different and the vowels of the first and second syllables were the same in half of the disyllabic pseudowords and different in the other half. Eighteen additional items with the same structure as the target pseudowords were used as no-go trials that were not analysed (see next section).

### Procedure

After equipping the participants with the EEG system, they were asked to sit in a sound-attenuated booth, approximately 70 cm away from the computer screen. The first task was explained to the participants. They were asked to produce the mono and disyllabic pseudowords in a delayed fashion. The participants could familiarize with the pseudowords by reading them once aloud from a list. In case they misread one of the pseudowords, the experimenter corrected their production. Then, participants received specific instructions, saw some examples, and underwent a training phase containing five warm-up trials similar to the experimental task. The experimenter guided the participants through the training. If necessary, the warm-up trials could be repeated. Once the participants felt comfortable with the task, the recording began.

**Fig. 1:**
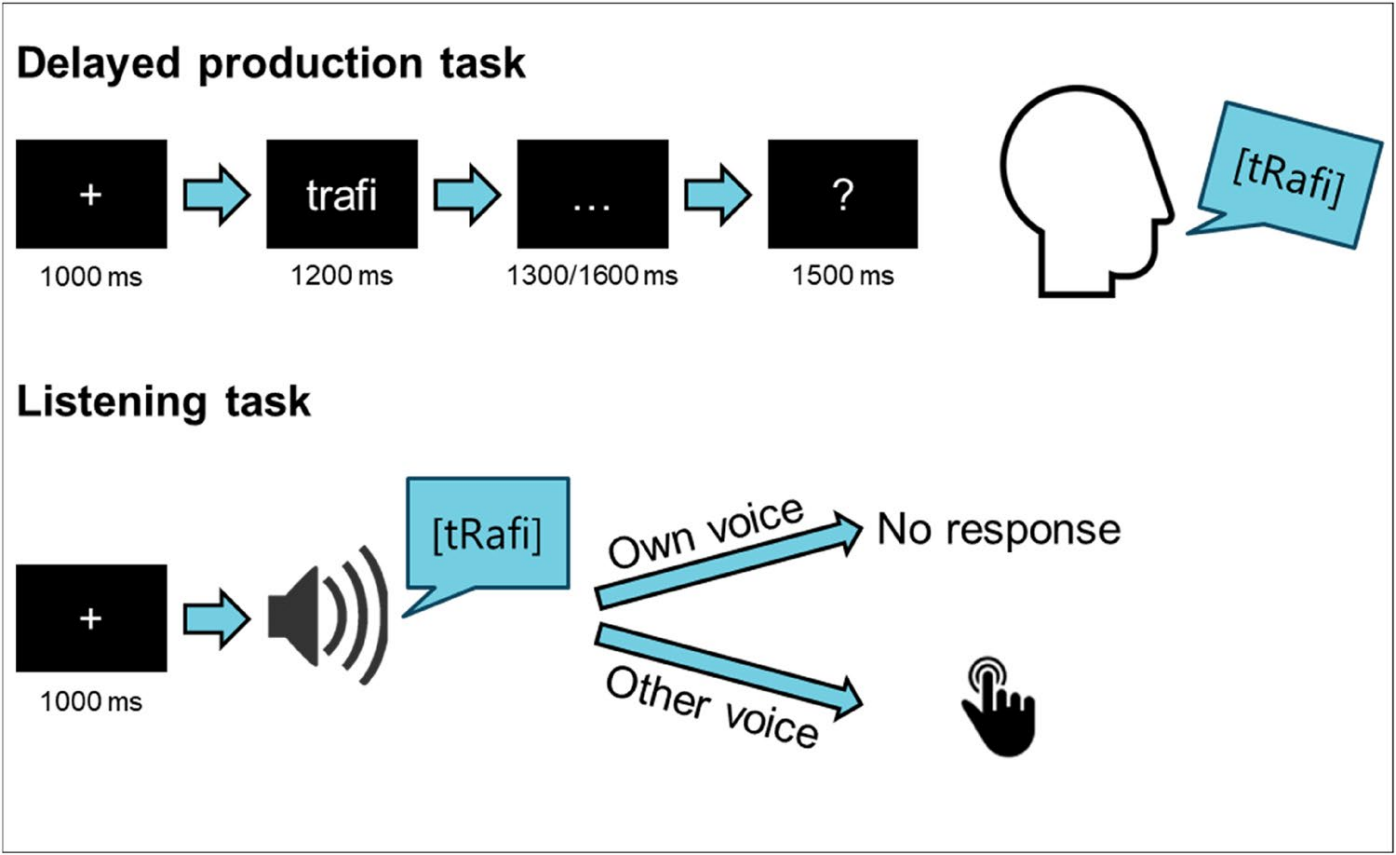
Experimental design. Delayed production task: Each trial consisted of a monosyllabic or disyllabic pseudoword appearing at the centre of the screen. The participants had to produce the pseudoword out loud as soon as the interrogation mark appeared on the screen. Listening task: The participants heard recordings of pseudowords, either their own productions recorded during the delayed production task, or rare productions from a person from the opposite sex. In the latter case, they had to press a key

Each experimental trial consisted of a “+” sign presented for 1000 ms, followed by the pseudoword for 1200 ms, then three dot appeared for either 1300 or 1600 ms, delaying the participants’ response. Finally, a response cue (question mark) prompted the participants to produce the pseudoword (Fig. 1, top). The duration of the delay was varied so that the participants could not anticipate when the response cue appeared. The no-go trials were presented randomly. In these cases, the response cue did not appear, and the participants had to remain silent until the next trial, starting after 500 ms.

Each disyllabic pseudoword was presented twice, once for each of the 1300 or 1600 ms delay lengths, resulting in 120 disyllabic stimuli. Monosyllabic pseudowords were presented six, eight, or ten times, corresponding to their occurrence in the disyllabic condition, resulting in 60 monosyllabic stimuli. Ultimately, the first task consisted of 198 stimuli (180 target stimuli and 18 no-go trials) divided into four blocks of 49 or 50 stimuli, allowing for short breaks during recording. Each participant received one of four different stimuli presentation order. Each order was pseudo-randomized, so that a given stimulus was not presented two times in a row, and so that the same delay was not presented in more than three consecutive trials.

For the second task, the participants were asked to listen to audio recordings of their own productions. The recordings were played through loudspeakers placed on each side of the screen. In this second part, 30 of their productions (50%) of each pseudoword was included, although for nine participants, all 60 monosyllabic productions were included. Eighteen recordings consisted of production from a person from the opposite sex. In this case, the participants were asked to press a response button. These trials were included in order to keep the participants focused on the task, and were not analysed.

The training and the tasks were completed using the E-prime software (version 3.0, Schneider et al., 2002), which recorded the behavioural data (audio files) collection. The continuous EEG signal was recorded throughout the experiment using the Active-Two Biosemi EEG system (Biosemi V.O.F. Amsterdam, Netherlands) with a 128-electrode cap.

### Acoustic Analyses and Behavioural Data Cleaning

For each trial, vocal onset was identified as the onset of the acoustic wave using the CheckVocal 2.2.6 software (Protopapas 2007). Response times were computed by measuring the delay between the appearance of the question mark and the vocal onset. We excluded from further analysis trials with response times shorter than 200 ms (anticipated responses) and trials with latencies exceeding two standard deviations from each participant’s mean. Trials with no responses or with production errors (i.e., phonemic deviation from the target syllable) were also discarded (mean: 2.8%; std: 3.9%).

In order to define the EEG analysis window, the acoustic duration of 50 monosyllabic trials selected randomly across participants was measured. The average duration was 307.6 ms with a high variability (SD = 55.4 ms). Thus, given the timing of both AEPs and uttering the first syllable, we set the EEG window of interest to 200 ms from vocal onset.

### EEG Acquisition and pre-processing

EEG data preprocessing was performed offline, using a customized MATLAB toolbox (De Pretto & Mouthon, 2023/2025). The processing pipeline is based on the Cartool software by Denis Brunet (github.com/DenisBrunet/Cartool), with additional EEGLab 2023.1 functions (Delorme and Makeig 2004) and in-built MATLAB functions. First, raw EEG data went through high- and low-pass Finite Impulse Response (FIR) filters removing frequencies below 0.5 Hz and above 40 Hz. Then, bridged electrodes were identified using the eBridge function (Alschuler et al. 2014). The number of bridged electrodes was unusually high most likely due to excessive use of gel. Indeed, while preparing the recordings, the signal showed high levels of noise on various channels probably due to wear and tear of the electrode sets. The data from eight participants were unusable and had to be removed from further analysis. Because most of the remaining participants still had problematic bridges, we reduced the number of channels from 128 to 68. We did so by removing every other electrode “row” of the original setting. The topographic maps of the AEP P1-N1 (Fig. 2) were consistent with the literature (Michel and Murray 2012), confirming the reliability of the ERPs. Even though such a procedure does not remove the bridges, it improves variability in the topographic maps, thus rendering the covariance matrices – at the basis of the linear filtering computation – more accurate. The fact that we used a repeated-measure design also limited the impact of the bridges in this exploratory study. Remaining bridges and bad channels identified during visual inspection in Cartool were interpolated (mean: 3.0 +/- 3.0%) using spherical splines (Perrin et al. 1989). The interpolation script used was written by Cohen (github.com/mikexcohen/AnalyzingNeuralTimeSeries). The signal was then re-computed to the average reference across the setting.

Event-related Potentials (ERPs) were computed for production and listening tasks by averaging epochs time-locked to the vocal onset. The epochs started 300 ms pre-vocal onset to include the time-window that has been associated with speech planning/programming (Laganaro 2023), and ended 200 ms post-vocal onset to focus on the articulation window of the first syllable. Epochs potentially contaminated by excessive noise and eye blinks were automatically identified if at least one channel presented a maximal voltage exceeding 80 µV. These epochs were either rejected or accepted after visual inspection.

For production, we computed different ERPs for the monosyllabic and disyllabic conditions, and ERPs comprising all types of pseudowords as markers of general production. The general production ERPs were used to compare the *AEP subtraction* and *linear filtering* versions of the ERPs to the original speech production ERPs. The individual ERPs included 51.4 ± 7.5 epochs in the monosyllabic condition and 50.5 ± 8.9 epochs in the disyllabic condition. The number of epochs was not statistically different between conditions. The disyllabic condition included only the pseudowords with different vowels between syllables, as these include more complex speech plans and motor programs, and thus increased the probability of observing differences between conditions. The general production ERPs included 152.4 ± 22.6 epochs. For the listening condition, we averaged together epochs from all types of pseudowords, as we were only interested in the signal associated with hearing the first syllable. This allowed to increase the quality of the signal used to simulate hearing oneself. The listening ERPs included 94.6 ± 19.7 epochs.

### Auditory Feedback Removal

#### AEP Subtraction

The individual AEPs from the listening task were subtracted from the individual production ERPs, channel per channel and timeframe per timeframe.

$$\:d\left[n\right]=\:y\left[n\right]-f\left[n\right]$$

where $\:d\left[n\right]$, $\:y\left[n\right]$, and $\:f\left[n\right]$ are $\:M$-dimensional vectors of samples at time index $\:n$ from the $\:M$ measured channels. $\:d$ represents the signal of interest $\:y$ is the production signal, and $\:f$ is the AEP signal.

#### Linear Filtering

The multi-channel Wiener filter is an optimal signal estimation method that leverages multiple input channels to minimize the mean squared error between the estimated and desired signals. Let us consider the $\:M$-dimensional vector $\:y\left[n\right]$ containing the signal at each of the $\:M$ channels at time index $\:n$. It is assumed that this vector is given by the superposition of the signal of interest $\:d\left[n\right]$ and the AEP signal $\:f\left[n\right]$ that we would like to suppress. The goal is to design an $\:M\times\:M$ dimensional linear filter $\:W$ that produces an estimate of the desired ERPs as.

$$\:\widehat{d}\left[n\right]={W}^{T}y\left[n\right]$$

The filter $\:W$ is chosen to minimize the mean-square error between $\:d\left[n\right]$ and $\:\widehat{d}\left[n\right]$. The optimal filter is then derived by using the covariance matrices of the signals as following.

We computed the estimated individual covariance matrices for both the speech production ERP and the listening AEP:

$$\:\widehat{R}=\frac{1}{N}Y{Y}^{T}$$

where $\:\widehat{R}$ is the estimated covariance matric, $\:N$ is the number of samples, and $\:Y$ is an $\:N\times\:M$ matrix containing the $\:N$ samples (of the speech production ERP or the listening AEP) at the $\:M$ channels.

We then subtracted the AEP covariance from the covariance of the production ERPs at the individual level, i.e.,

$$\:{\widehat{R}}_{dd}={\widehat{R}}_{yy}-{\widehat{R}}_{ff}$$

where $\:{\widehat{R}}_{yy}$ is the estimated covariance matrix of the original production signal, $\:{\widehat{R}}_{ff}$ is the estimated covariance matrix of the listening signal, and $\:{\widehat{R}}_{dd}$ is the estimated covariance matrix that theoretically represents the production signal without the auditory feedback.

We can then obtain the optimal filter $\:W$ that can be used to estimate the signals of interest without the auditory feedback AEP as:

$$\:W={{\widehat{R}}_{yy}}^{-1}{\widehat{R}}_{dd}$$

We recomputed the ERP to which the AEP covariance was removed:

$$\:D={W}^{T}y\left[n\right]$$

Spatial covariance of EEG channels represents the coherence between the channels and thus, reflects patterns of brain activation (Gopan et al. 2022; Ju et al. 2023). It has notably been used for classification of specific EEG profiles (e.g., Congedo et al. 2017; Gopan et al. 2020). Because the AEP is composed of multiple ERP components reflecting different steps on the processing of auditory perception, we computed covariance for each component separately. Thus, the first step was to identify the APE components. For this, we performed a microstates decomposition (Brunet et al. 2011; Lehmann et al. 1987; Murray et al. 2008) using the RAGU software (Koenig et al. 2011) and forcing the software to identify two maps. This allowed to identify the first two typical components (Fig. 2). The P1 component ranged from 40 ms to 92 ms, and the N1 component ranged from 93 ms to 172 ms. These latencies are later than what is usually measured in AEPs (Legatt 2015) because the vocal onset set as the origin of the AEP corresponded to the initial voiceless plosive consonant, while the AEP was most likely aligned to the following liquid phoneme consonant.

Finaly, in the original production ERPs, we replaced the original signal of these periods with the corrected signal. Since there is no explicit amplitude preservation constraint in the formulation of the multichannel Wiener filter, the GFP values of the resulting ERPs might differ from those of the original ERPs. Therefore, we normalised the signal according to the GFP of the original production ERP. Specifically, for each period, we normalised the signal at each timeframe according to the GFP value of the first timeframe of this period in the original signal. This procedure maintained the scalp topographies of the new signal. However, at the channel level, some jumps in amplitude could still be observed, so we used a moving mean over eight timeframes (16 ms), from − 16 ms to + 16 ms around the transition. We tested different values for the moving mean size (including no moving mean), and all yielded the same microstate analysis results. Thus, this step served only for visual presentation. The MATLAB script used for linear filtering is provided in the supplementary material.

### EEG Data Analyses

The different versions of the signal were assessed using EEG microstate analyses in order to identify potential modulations in field strength or topographic maps during sustained periods of time and whether these modulations depended on the version of the signal. Firstly, we compared all three versions of the general production signal using one-way repeated measures ANOVAs (original production signal; subtraction of AEP; AEP linear filtering). Then, we compared Mono- versus Disyllabic conditions for the original production signal and for the AEP linear filtering version in two separate analyses.

We analysed the ERPs using global, data-driven procedures including global field power (GFP), global map dissimilarity (GMD), and microstates (Habermann et al. 2018). These procedures are reference independent and take into account the EEG signal in its entirety. For microstate analysis, we considered Holm-Bonferroni corrected effect of map duration, area under the curve (AUC), mean GFP, and centre of gravity. Moreover, microstate effects were considered if they were associated with GFP and/or GMD effects of at least 30 ms.

All EEG data analysis were conducted using the RAGU toolbox (Koenig et al. 2011) by computing 5000 timeframe-wise randomisation statistics. The alpha threshold was set at 0.05. Before analysing the data per se, a topography consistency test (Koenig and Melie-García 2010) allowed to assess whether the patterns of neural activation were consistent across participants.

## Results

**Fig. 2:**
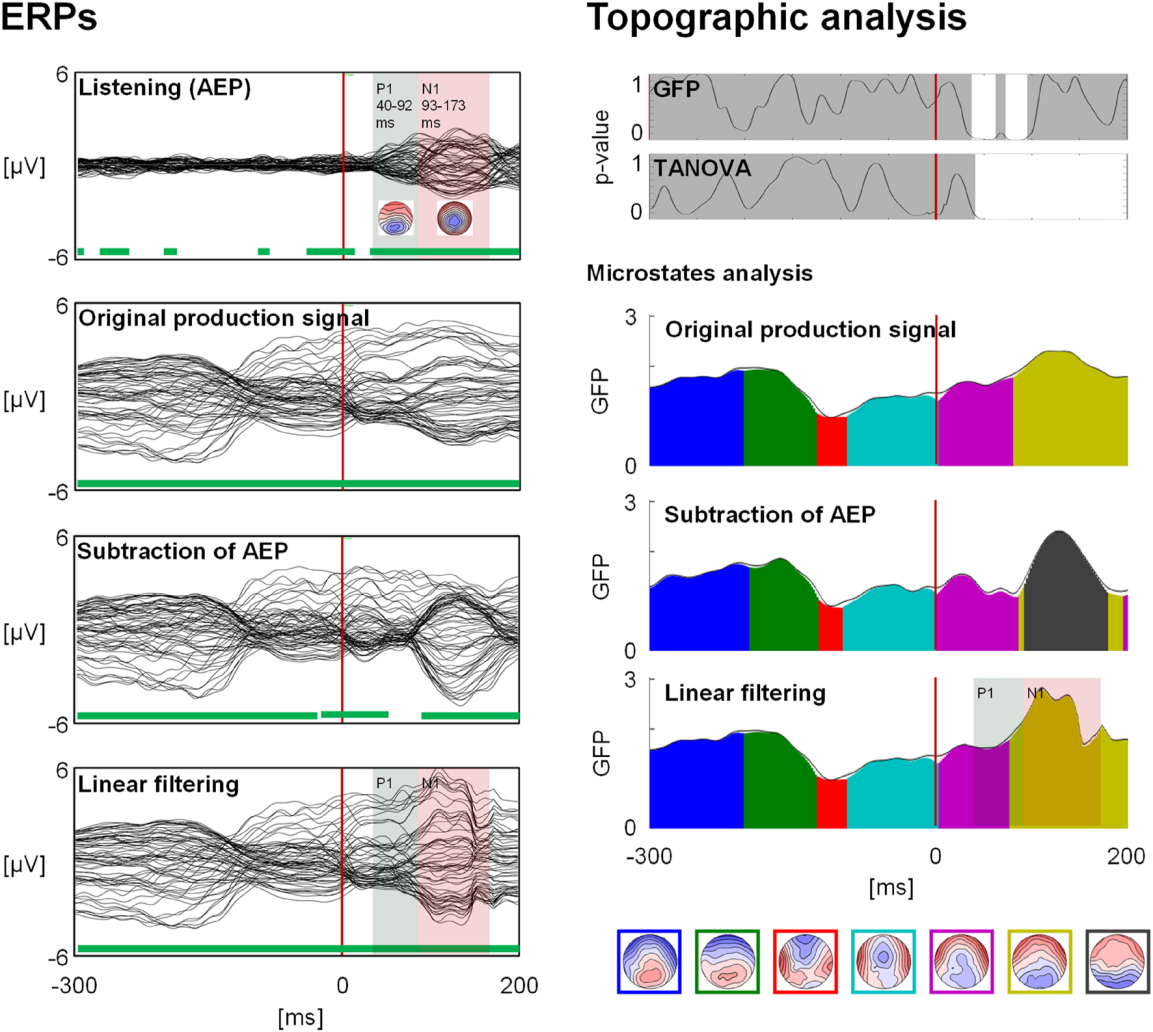
General production analysis.** Left**: Grand-average ERPs. The first ERP (top) depicts the AEP of the listening task. The shaded green and red periods represent AEP components that were used for linear filtering. Below are the three version of the speech production signal. The green lines below each ERP indicate periods of topography consistency. The red vertical lines indicate vocal onset (0 ms). **Right**: Topographical analysis of the three version of the signal, showing post-vocal onset difference in the subtraction version only. In the GFP and TANOVA graphs, the white periods indicated periods of statistically significant differences between versions. These periods are all longer than 20 ms. The microstates analysis shows an additional map for the subtraction of AEP version (black). The red vertical lines indicate vocal onset (0 ms). For the linear filtering versions, the shaded green and red periods represent the periods of the AEP components that were processed

### General Production Analysis

Topography consistency tests indicated consistent patterns of neural activation across participants for the original production signal and the AEP filtering versions (Fig. 2, left, green lines). The subtraction of AEP version showed a period of topographic inconsistency in a time window corresponding roughly to the AEP P1 component.

The GFP analysis yielded two periods of significant differences between conditions, running from 38 ms to 61 ms, and from 73 ms to 95 ms. The TANOVA analysis yielded one period of significant differences between conditions, running from 42 ms to 200 ms.

Microstate analysis comparing all three versions of the signal yielded an additional microstate (black map, Fig. 2) for the AEP subtraction version. This effect was confirmed by the TANOVA effect. The time window in which this map appeared (95 ms to 179 ms) corresponded to the AEP N1 component. All other map indices were similar between the three versions of the signal.

**Fig. 3:**
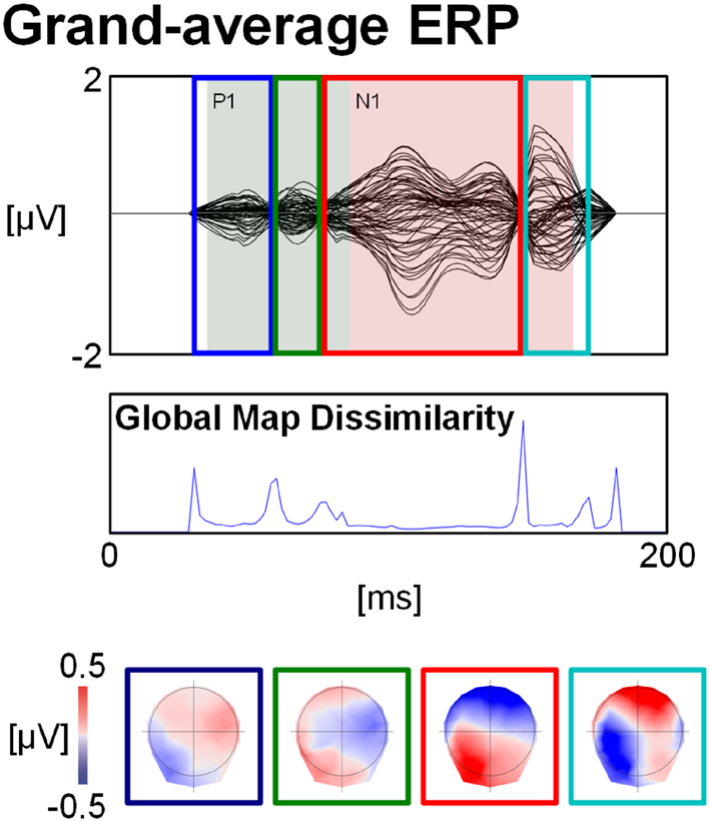
Signal removed by linear filtering. The waveforms depict the signal of the channels obtained by subtracting the linear filtering grand average from the original production grand average. Shaded green and red periods represent AEP components. The peaks of the Global Map Dissimilarity index were used to decomposing the signal into periods of topographic stability. These topographies are shown at the bottom, also indicating the intensity of the signal removed by linear filtering.

Figure 3 depicts the activity removed by linear filtering. The post-vocal onset ERP represents the subtraction of the signal after the linear filtering from the original signal. Each AEP component was divided into two periods of topographic stability. These topographies differed from the classical AEP components (Fig. 2)

### Monosyllabic Versus Disyllabic

**Fig. 4:**
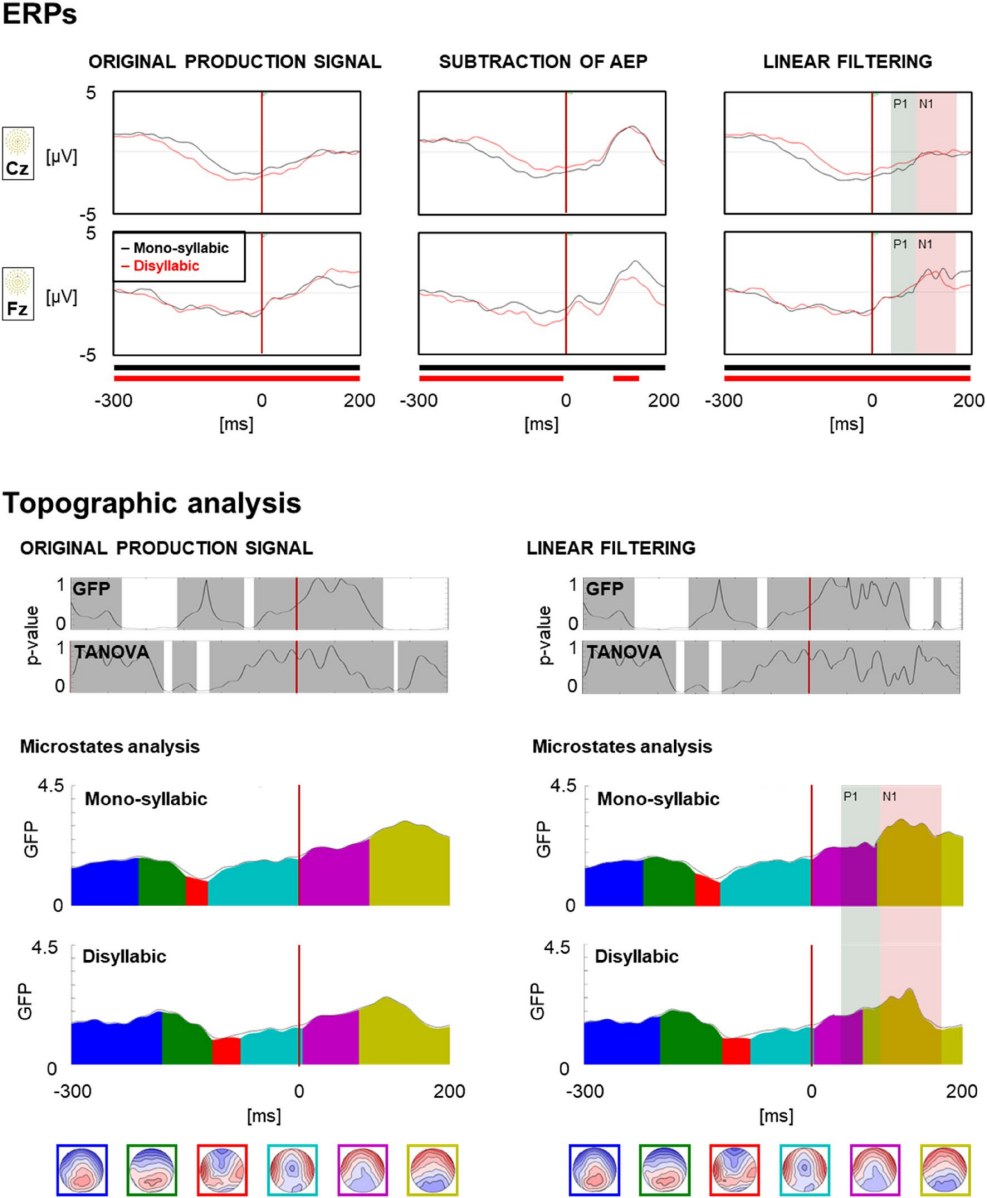
Comparing Mono- versus Disyllabic conditions**. Top**: Grand-average ERPs for each version of the signal. The waveforms compare the mono-syllabic (black) and disyllabic (red) conditions for two channels (Cz and Fz). The lines below the ERPs indicate periods of topography consistency for mono-syllabic (black) and disyllabic (red) conditions. The red vertical lines indicate vocal onset (0 ms). For the linear filtering versions, the shaded green and red periods represent the periods of the AEP components that were processed. **Bottom**: Topographical analysis of the original production signal (left), and the linear filtering (right) versions. In the GFP and TANOVA graphs, the white periods indicated periods of statistically significant differences between conditions. The red vertical lines indicate vocal onset (0 ms). For the linear filtering versions, the shaded green and red periods represent the periods of the AEP components that were processed. The AEP subtraction version is not included due to inconsistent topographies

Topography consistency tests indicated consistent patterns of neural activation across participants in both conditions of the original production signal and the linear filtering versions (Fig. 4). Moreover, these signals show Bereitschaftspotential components at Cz replicating previous results of speech production (Chandregowda et al. 2020; McArdle et al. 2009). The subtraction of AEP version showed two large periods of topographic inconsistency following vocal onset in the disyllabic condition. Thus, this version of the signal was not further analysed.

For the original production signal, the GFP analysis yielded three periods of significant differences between conditions, running from − 232 ms to −159 ms, from − 70 ms to −58 ms, and from 114 ms to 200ms. The TANOVA analysis yielded three periods of significant differences between conditions, running from − 175 ms to −165 ms, from − 132 ms to −116 ms, and from 130 ms to 134 ms. For the AEP linear filtering version, the pre-vocal onset time periods yielded the same results as for the original production signal version, as this part of the signal was not processed during linear filtering. Post-vocal onset, the only difference was that the GFP analysis yielded two periods of significant differences between conditions, instead of only one, running from 132 ms to 170 ms, and from 173 ms to 198 ms.

The microstate decomposition yielded similar results on the original production signal and on the linear filtering versions. Detailed results are reported in Table 1. The differences were mainly identified pre-vocal onset. Combined with the GFP results, this effect concerned mainly the centre of gravity of Map 2, reflecting a delayed processing in the disyllabic condition.

**Table 1:** Comparisons (p-values Holm-Bonferroni corrected) between conditions (monosyllabic vs. disyllabic) on each microstate map in the original signal and after linear filtering

	Map 1	Map 2	Map 3	Map 4	Map 5	Map 6
ORIGINAL PRODUCTION SIGNAL						
Duration:	0.046	0.849	0.583	0.075	0.091	0.261
AUC:	0.094	0.877	0.643	0.044	0.149	0.357
Mean GFP:	0.851	0.952	0.740	0.111	0.220	0.608
Centre of gravity:	0.046	0.005	0.059	0.071	0.586	0.210
LINEAR FILTERING						
Duration:	0.083	0.421	0.783	0.075	0.170	0.316
AUC:	0.100	0.491	0.936	0.038	0.192	0.432
Mean GFP:	0.956	0.866	0.493	0.110	0.172	0.311
Centre of gravity:	0.086	0.009	0.039	0.077	0.572	0.285

## Discussion

The aim of this study was to assess the use of a linear statistical filtering technique as a way of suppressing the part of the signal associated with auditory feedback from speech production ERPs in order to test whether AEP masks other cognitive-motor processes going on in parallel during speech articulation. For this purpose, we compared three versions of the ERPs. The first version was the *original production ERP*, with no further processing other than classical ERP preprocessing and averaging. The second version was the *AEP subtraction ERP*, where the ERP from the listening task was subtracted from the production ERP. The third version consisted of the *linear filtering ERP*, where the signal of interest was retrieved from the production ERP by supressing an estimate of the unwanted noise induced by the AEP associated to auditory feedback.

### Subtraction of AEP

When comparing the three versions of the signal, AEP subtraction showed a specific microstate map that was not present in the other two versions. The latency of the map corresponded to the N1 AEP component. Taken alone, this new map is difficult to interpret, as removing the listening AEP from the production ERP does not guarantee that the new map will have any physiological meaning. However, the fact that AEP subtraction ERPs showed periods of topographic inconsistency (Koenig and Melie-García 2010) suggests that, as expected, subtraction was inappropriate for isolating speech planning/programming brain processing from the auditory part of the signal. Further supporting this conclusion, comparing mono- versus disyllabic conditions yielded even longer periods of topography inconsistency.

In the general production analysis, the period where topography consistency is not met occurs roughly during the AEP P1 component (Fig. 2). The topography of this component is very similar to the topography of Map 5 occurring at the same time during speech production. Thus, the subtraction may have brought the GFP closer to 0, which in itself is a sign that there is no consistent underlying brain source, and which is unlikely at this latency. Moreover, the subtraction might increase the variance of the between-participant maps, as the most stable part of the signal will be removed and the noise will remain. In other words, it will reduce the signal-to-noise ratio and thus result in the obtained TCT output.

Regarding the mono- versus disyllabic analysis, a similar explanation is possible. However, the inconsistent topography periods were only bound to the disyllabic condition, suggesting that the reason for this result may be more complex. One explanation could be that some delayed processing or parallel ongoing encoding processes during the production of longer speech sequences (disyllabic) as observed in the pre-vocal onset signal (see results section and next section) might still take place, even though not statistically significant post-vocal onset. Thus, the signal during disyllabic production may vary greatly between participants, whereas the AEP signal is more constant.

Regarding the reasons why the AEP of the listening is not reproducing what happens during auditory feedback, three factors might have accentuated the difference between the N1-P1 AEP components between the listening and the production tasks. Firstly, attention towards the audio recordings might have increased the amplitude of the AEP in the listening task (Picton and Hillyard 1974). However, the task required minimal attentional resources, as deviant trials were obvious (own voice versus voice of someone from the opposite sex). Secondly, the N1-P1 AEP components have been shown to react to distortions of one’s own voice during auditory feedback, associated with violation in expectancy (Scheerer and Jones 2018). Again, this impact might have been limited. Even though hearing a recording of one’s own voice differs from auditory feedback during speech production, this was expected and constant throughout the task. Finally, the inhibition of the auditory cortex during speech production appears as the most probable reason justifying that the listening AEP signal differs from the auditory feedback signal during articulation.

### Linear Filtering

Regarding linear filtering, it showed a microstate pattern similar to that of the original production ERP, indicating that such an approach preserved the general scalp topographies of the signal. The signal removed by linear filtering showed more than the two topographies classically associated with AEPs (Fig. 2 and Michel and Murray 2012), confirming that linear filtering does more than simply removing the AEP, and suggesting that what happens during auditory feedback involves cognitive processes more complex than during listening a recording of one’s own voice.

Consequently, the mono- versus disyllabic contrasts based on the original production signal and the linear filtering yielded similar results. Even though the microstate results were not accompanied by strong TANOVA results, the general pattern suggests delayed processing preceding the vocal onset when planning disyllabic pseudowords as opposed to monosyllabic pseudowords, a result that is coherent with the amount of speech to be planned in each condition.

Linear filtering did not reveal additional/hidden differences across the two conditions contrasted here (mono-syllabic versus disyllabic speech production), suggesting that it may be unnecessary to remove auditory feedback signal when the overlapping of signals is balanced between conditions (here the same first syllable in the exact same delayed production task). Nevertheless, linear filtering may be useful beyond its current use in EEG artefact removal. Distinguishing overlapping cognitive components from one another is of great interest in the literature (e.g., Ehinger and Dimigen 2019; Ouyang et al. 2016; Ross et al. 2022). Many solutions have been developed. However, most of these require temporal jittering between the components to be separated, which is not the case of linear filtering. This does not mean that it may be useful only when the overlap is complete, and it may help in situations where the temporal delay between the signals of interest is too small for the existing solutions to be efficient. Even though the current results failed to extract potentially hidden differences between mono- and disyllabic conditions, it demonstrated the usability of the MWF in ERP contexts.

### Mono- Versus Disyllabic Processing

Here, the lack of differences between monosyllabic and disyllabic conditions suggests that planning/programming disyllabic speech sequences may be achieved before the production of the first syllable. It may be that the bridges between electrodes could have blurred the topographic maps and thus reduced sensitivity. However, our results are consistent with recent evidence that motor planning/programming occur before vocal onset (Bullock et al. 2025; Dorokhova et al. 2024). Alternatively, speech planning/programming of subsequent syllables may not be time locked to the onset of the first syllable, and thus may have cancelled out during averaging. However, to be fully cancelled out, these processes should be sufficiently jittered relative to vocal onset, which is unlikely given the short production time of disyllabic words. Nonetheless, this hypothesis could be verified by comparing disyllabic conditions and analysing the data time-locked to the onset of the second syllable.

It may be possible to study speech planning/programming by designing studies with conditions limiting auditory feedback. For example, using pink noise to mask auditory feedback, Christoffels et al. (2007) recorded fMRI during a picture naming task and could demonstrate inhibition of the auditory cortex during speech production. Other studies have tested imagined speech (e.g., de Borman et al. 2024; Proix et al. 2022), tough this implies the absence of motor activation, making it unlikely to represent speech planning and programming. To address this issue, it may be possible to ask participants to mouth words without producing sounds. In this case, only a subset of muscles will be activated. Nonetheless, this could provide significant insights into speech production during articulation. Although relevant and complementary, these methods lack the ecological validity of studying overt speech production. Soroush and collaborators (2023) recorded intra-cranial EEG during overt, mouthed, and imagined sentence reading. They showed unique neural networks to each mode, cautioning against studying one mode as a surrogate for another.

In conclusion, following the approach of minimally intervening on the data (Delorme 2023), it is probably unnecessary to remove auditory feedback or other overlapping signal if the signals are time-locked to each other. Moreover, signal that may serve as a template for the MWF, such as the listening condition in the current study. This implies to prolong the recording sessions, and potentially reduce the number of trials of the main task, which might reduce the quality of the ERPs by reducing the number of averaged epochs. However, linear filtering may still be useful in cases where the temporal jitter between the overlapping signals is too weak for other methods to be efficient.

## Appendix A1 - Pseudowords

**Table:** 

Monosyllabic
kla
kra
krou
plou
pra
prou
tra

**Table:** 

Disyllabic	
klacha	klachi
klafa	klafi
klapa	klapi
klata	klati
krafa	krafi
krapa	krapi
krassa	krassi
krata	krati
krouchou	krouchi
kroufou	kroufi
kroussou	kroussi
kroutou	krouti
plouchou	plouchi
ploufou	ploufi
ploukou	plouki
ploussou	ploussi
ploutou	plouti
pracha	prachi
prafa	prafi
praka	praki
prassa	prassi
prata	prati
prouchou	prouchi
proufou	proufi
proukou	prouki
proussou	proussi
proutou	prouti
tracha	trachi
trafa	trafi
trapa	trapi

## Supplementary Information

Below is the link to the electronic supplementary material.

Supplementary Material 1

## Data Availability

The data that support the findings of this study are available on request from the corresponding author. The data are not publicly available due to ethical restrictions.
